# Six Years’ Experience With a Novel Dissection-Specific Stent-Graft to Prevent Distal Stent-Graft-Induced New Entry

**DOI:** 10.1177/15266028241257106

**Published:** 2024-05-31

**Authors:** Maysam Shehab, Anders Wanhainen, Gustaf Tegler, David Lindstrom, William Yoon, Kevin Mani

**Affiliations:** 1 Section of Vascular Surgery, Department of Surgical Sciences, Uppsala University, Uppsala, Sweden; 2 Faculty of Medicine, Tel-Aviv University, Tel-Aviv, Israel; 3 Division of Surgery, Department of Surgical and Perioperative Sciences, Umeå University, Umeå, Sweden; 4 Division of Vascular Surgery and Endovascular Therapy, University Hospitals Harrington Heart & Vascular Institute, Case Western Reserve University, Cleveland, OH, USA

**Keywords:** dissection-specific stent-graft, distal stent-graft-induced new entry, chronic type B aortic dissection

## Abstract

**Introduction:**

Thoracic endovascular aortic repair (TEVAR) in chronic dissection is associated with a risk for distal stent-graft-induced new entry (dSINE) in up to a quarter of cases. We assess the mid-term outcome of a novel dissection-specific stent-graft (DSSG), which is a custom-made device based on the Cook Alpha platform, with a modified graft design and a distal endovascular elephant trunk without any supporting stent to reduce the radial force on the dissection membrane at the distal landing zone.

**Methods:**

A retrospective single-center study of chronic dissection patients at high risk of dSINE who received an elective endovascular repair with DSSG from January 2017 to June 2023. The primary outcome is Kaplan-Meier (KM) estimated freedom from dSINE during follow-up. Secondary outcomes included technical success, aortic remodeling, and anatomical evaluation of the distal landing zone in cases with dSINE during follow-up versus those without.

**Results:**

Thirty patients (24 males) with a median age of 66 years [range=31-78] underwent elective TEVAR with the DSSG. The majority, n=27 (90%), had previous aortic repair; 7 (23%) had established connective tissue disease, and 6 (20%) had established dSINE after previous stent-graft implantation as an indication for TEVAR. Technical success was achieved in n=29 (97%). Median follow-up was 38.5 months (4.3-76.4), and KM estimated freedom from dSINE at 1 and 3 years was 95.6% (SE 0.043) and 89% (SE 0.081), respectively Four cases developed dSINE during follow-up. The median distance from the distal stent-graft to the coeliac trunk was 74mm (range=18-123) in the dSINE group versus 26mm (range=0-74) in the non-dSINE group (p=0.049). Median proximal tangential aortic angulation in the distal landing zone was 38.5° (range=26°-50°) in the dSINE group compared to 21° (range=3-61°) in the non-dSINE group (p=0.052).

**Conclusions:**

The Use of a novel DSSG with low radial force for TEVAR in the setting of chronic dissection is safe and feasible, with remodeling outcomes comparable with standard TEVAR. The reduced distal radial force in the DSSG does not eliminate the risk for dSINE over time, with new entries occurring, particularly in cases where the distal landing zone is in a tortuous aortic segment and not close to the coeliac trunk.

**Clinical Impact:**

Using the novel dissection-specific stent-graft with reduced radial force is safe and feasible but does not completely eliminate the risk of dSINE occurring over time. The exact positioning of the distal stent-graft in a straight aortic segment, close to the coeliac trunk, may be of importance to further mitigate the risk.

## Background

Thoracic endovascular aortic repair (TEVAR) is increasingly used to manage chronic dissection aneurysms. In a recent international analysis performed by the Vascunet collaboration, some 43% of TEVAR procedures for type B aortic dissection are performed in the chronic phase.^1^

Descending thoracic aortic coverage with a stent-graft aims to result in thrombosis of the false lumen along the stent-graft and remodeling of the thoracic aorta, with complete thrombosis occurring in 30% to 51% of cases.^2–4^ However, implantation of a stent-graft in the true lumen of a dissected aorta is associated with the risk for stent graft-induced new entry, defined as a new tear caused by the stent graft itself, excluding those created by natural disease progression or any iatrogenic injury from endovascular manipulation. These tears may be proximal, leading to pseudoaneurysm formation or retrograde dissection, or distal, resulting in false lumen pressurization and expansion by type IB entry flow to the false lumen (so-called distal stent graft-induced new entry or dSINE).^5^ In chronic aortic dissection, the dissection membrane has often stiffened over time, and the potential for remodeling is lower. Therefore, the shear stress between the distal end of the stent graft and the dissection membrane is high and may cause dSINE.^6^

Previously observed incidence rates for dSINE showed significant variation, from 5% to 28%.^6–9^ Comparative morphological analyses of patients post TEVAR with and without dSINE have identified small true lumen diameter, a more accentuated oval postoperative stent graft morphology, high degree of oversizing, severe aortic angulation, and type III aortic arch as risk factors for dSINE development.^6,9^

In the chronic dissection setting, the size of the true lumen in the distal landing zone is often smaller than the proximal landing zone, resulting in a greater radial force of the stent-graft on the distal aortic wall. While the concept of dSINE is commonly recognized after TEVAR in aortic dissection, graft-induced new entry was not reported after standard surgical elephant trunk technique where a nonsupported polyester graft is inserted in the true lumen, suggesting that one risk factor for dSINE is the radial force of the stent-graft against the dissection membrane.

**Figure 1.**
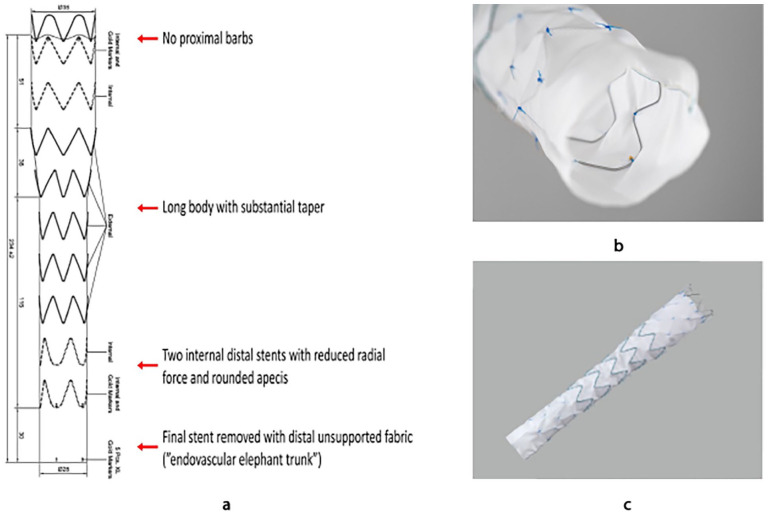
(a) Schematic drawing showing the main technical features of the custom-made dissection-specific stent-graft which is based on the Cook Alpha thoracic stent-graft platform; (b) Visualization of the distal internal stents with reduced radial force and the distal unsupported fabric; (c) Overview image of the dissection-specific stent-graft.

A novel dissection-specific stent-graft (DSSG) that is custom-made, based on the Cook Alpha thoracic endograft platform (Cook Medical Inc, Bloomington, IN, USA), may be an alternative in this situation. This device aims to remove the distal radial force of the stent-graft against the dissected aortic membrane. This stent-graft includes several modifications, including substantial tapering, 30% to 60% reduction in radial force of the distal stents, and removal of the final stent, allowing for an unsupported endovascular elephant trunk at the distal end of the stent graft with minimum radial force, Figure 1.^7,8^

The short-term results of using this stent-graft have previously been reported by our team. The aim of this study is to assess the outcome of this novel device in an expanded cohort with longer follow-up.

## Materials and Methods

A retrospective single-center cohort study of patients with chronic aortic dissection (>90 days from inciting event) who received an elective endovascular repair with the DSSG from January 2017 to June 2023. The indication for intervention for chronic dissection at the center is maximal aneurysm diameter ≥ 5.5 cm in low-risk patients or ≥6 cm in high-risk individuals or growth rate ≥1.0 cm over 12 months.

The DSSG was employed in all cases deemed high risk for dSINE, including patients with connective tissue disease and those with already established dSINE after previous stent-graft implantation in the descending aorta. The follow-up protocol included computed tomography angiography (CTA) imaging at 1 month and annually thereafter. The details of the TEVAR procedure have previously been reported.^8^

### Data Collection and Outcomes

The study’s primary endpoint is dSINE occurrence on follow-up CTA at any time after the index intervention. The definitions of data variables were based on the SVS STS Reporting Standards for Type B Aortic Dissections.^5^ Baseline characteristics, imaging, intervention details, complications, and death were retrieved from the patient’s electronic records. Secondary outcomes included early (<30 days) technical success as well as early complications, including spinal ischemia (permanent or temporary), cerebrovascular events, cardiovascular events, unplanned reinterventions, overall mortality, and aortic mortality. Technical success was defined as successful deployment of the stent-graft with complete coverage of the primary entry tear and no signs of type 1 endoleak, unintentional supra-aortic trunk occlusion, conversion to open surgery, or retrograde type A dissection. Unplanned re-intervention (early <30 days) was defined as unplanned secondary surgical procedures required to correct procedure-related adverse events and/or graft-related complications (migration, endoleaks, occlusion, collapse, infolding, or fracture). Late outcomes (>30 days) included aortic remodeling, unplanned reinterventions, overall mortality, and aortic mortality.

For imaging analysis, CTA three-dimensional multi-planar and centerline analysis were performed using 3Mensio Vascular software (3Mensio Medical Imaging B. V., Bilthoven, The Netherlands). Any new occurrence of dSINE was noted on all follow-up imaging. Proximal landing zone was evaluated based on the Ishimaru classification.

While all patients contributed to the early outcomes and survival analysis, patients were censured from long-term follow-up regarding the occurrence of dSINE if there was a technical failure, death within 30 days, or no CTA was available for review after the index procedure. Follow-up ceased if patients received further distal interventions (TEVAR, FEVAR, or open repair, which precluded the development of dSINE).

Aortic measurements included maximal descending aneurysm diameter, coverage length, and distance from the distal end of the DSSG and the top of the coeliac trunk (CT). When measuring the aortic diameter and the diameter of the true and false lumen, the average value of 2 perpendicular measurements was used in the case of an elliptical true lumen shape.

The length was measured with aortic centerline analysis. Aortic remodeling was defined as aneurysm sac behavior (at the level of maximal diameter with outer wall to outer wall measurements) in *the treated* segment. It was classified on a 3-point scale as decreased, stable, or increased (absolute increase or decrease ≥ 5 mm compared with preoperative diameter). In addition, false lumen thrombosis at the level of the covered aorta was also evaluated (based on the presence of contrast in the false lumen on arterial-phase or delayed contrast-enhanced imaging).

**Figure 2.**
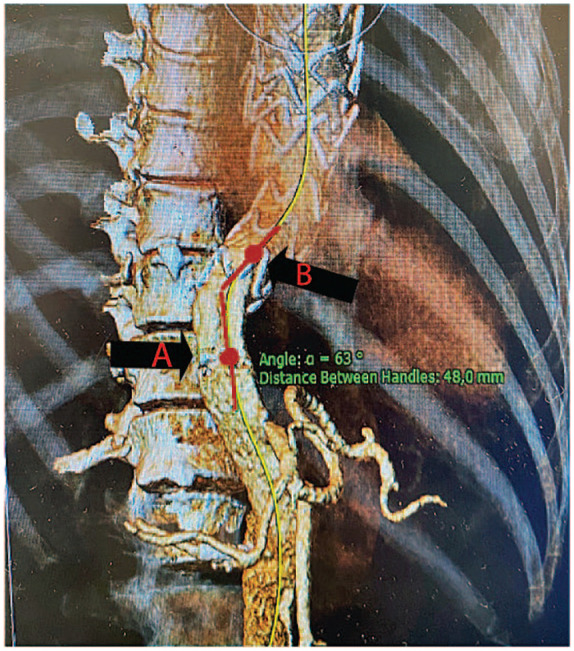
The angulation of the aorta at the distal landing zone of the thoracic stent graft was evaluated using the tangent angle tool within the 3mensio software. Defined as the tangent line and the vessel axis at a specific point along a blood vessel. The first point (A) is the distal end of the stent-graft, the second point (B) is on the next proximal straight aortic segment accordingly, the distance between the 2 points is <5 cm.

The aortic angulation at the distal landing zone was evaluated by measuring the tangent angle at the distal stent graft using a specific tool in the 3mensio software, Figure 2. The measurement process involved the following steps: first, the aortic centerline was extracted from the 3D reconstruction images. The point on the centerline that delimits the transition from covered to uncovered aorta at the distal end of the stent-graft was marked (point A). Subsequently, a second point (B) was marked on the proximal straight, stented part of the aorta. The distance between the 2 points may not exceed 50 mm. The angle formed between the tangent lines at point (A) and point (B) captures the vessel trajectory; the larger the angle, the sharper the curve.

The study was approved by the local ethics committee, which waived the need for written informed consent for this retrospective analysis.

### Statistical Analysis

Data are expressed as median and range for continuous variables. Categorical variables are presented as numbers and percentages. Univariate analyses were evaluated using the Mann–Whitney U test for nonnormally distributed continuous variables and Chi-square or Fisher’s exact test for categorical variables. A p-value <0.05 was considered statistically significant.

Data were analyzed using IBM SPSS version 29 (BM Corporation, Armonk, NY, USA).

## Results

Thirty patients (24 males, 6 females) with a median age of 66 years (range=31-78) underwent elective TEVAR using the DSSG. During the same period, some 312 cases of thoracic endovascular aortic stentgraft implantations were performed for various aortic pathologies, and the DSSG hence constituted 10% of these cases. The median follow-up duration was 38.5 months (range=4.3-76.4). Patient characteristics are presented in Table 1. Of note, 7 patients had a verified connective tissue disorder, six (20%) had an established dSINE after previous aortic procedures (4 cases of previous TEVAR, 2 cases of previous Frozen Elephant Trunk (FET)), and the DSSG was inserted as a distal extension of the existing repair, while in the remaining 24 (80%) patients the DSSG was used as primary treatment for CTBAD-related aneurysms.

**Table 1.** Baseline Characteristics of Patients With Chronic Aortic Dissection Undergoing Dissection-Specific Stent Graft Implantation (N=30).

	N (%) or median (range)
Age (years)	66 (31-78)
Gender	
Female	6 (20%)
Male	24 (80%)
Diabetic Miletus	3 (10%)
Hypertension	26 (86.7%)
Lung disease	5 (16.7%)
Cardiac disease	15 (50%)
Renal insufficiency	4 (13.3%)
Connective tissue disorder	7 (23%)
- Marfan	5
- Loeys-Dietz syndrome	2
Prior aortic repair	27 (90%)
Indication for repair	
- Aneurysm	24 (80%)
- dSINE	6 (20%)

dSINE, distal stent-graft-induced new entry tear.

Two patients were excluded from operative detailing and the long-term outcome analysis (<30-day death and technical failure). Fourteen patients (50%) had Ishimaru’s zones 3 and 4 as a proximal landing zone. For those landing in zone 4, the prior repair ranged from zone 0 to 3. Therefore, the treatment was confined to the descending thoracic aorta, either by placing DSSG alone or in combination with proximal thoracic stent-graft using a standard commercially available thoracic endograft platform (Gore C-TAG or Cook Zenith TX2). Arch vessels revascularization (hybrid arch debranching, FET, parallel stent graft, and complete endovascular branched reconstruction with branched devices) was performed in all the remaining fourteen (50%) patients to achieve a safe proximal landing zone in Ishimaru’s zone 0-2.

The median DSSG length was 202.5mm (range=122-234), and the proximal and distal stent-graft median diameter was 34 mm (range=28-44) and 28mm (range=22-38mm), respectively. The median tapering ratio of the DSSG was 22.2% (range=0%-36.4%), the median difference between the proximal and distal diameter of the DSSG was 8mm (range 0-16mm), and the median proportion of the descending aorta covered with a TEVAR graft was 89.5% (range=59.9%-100%).

The median tangent angle at the distal landing zone was 24° (range=3°-61°), and the median distance between the distal end of the stent-graft to the CT was 27mm (range=0-123). The median distal landing zone diameter was 20mm (range=14-29). The median graft oversizing in the distal landing zone was 33.3% (range=5.7%-64.7%), Table 2.

**Table 2.** Operative Details of Patients With Chronic Aortic Dissection Undergoing Implantation of a Dissection-Specific Stent-Graft (N=28).

	*N*^a^ (%) or median [range]
Proximal landing zone^b^	
zone 0	8 (29%)
zone 1	2 (7%)
zone 2	4 (14%)
zone 3	4 (14%)
zone 4	10 (36%)
Graft length (mm)	202 [122-234]
Proximal graft diameter (mm).	34 [28-44]
Distal graft diameter (mm).	28 [22-38]
Taper ratio (%)	22 [0-36]
Distal landing zone diameter, (mm)	20 [14-29]
Distal oversizing (%)	33.3 [5.7-64.7]
Descending aorta coverage (%)	89 [60-100]
Tangent angle (degrees)	24 [3-61]
Distance from distal graft to coeliac trunk (mm)	27 [0-123]

a Two patients were excluded from this analysis (1 case of 30-day death, 1 case of technical failure). ^b^ Ishimaru’s zone.

Technical success was achieved in all but one case, as the true lumen was still collapsed; this required distal extension across the celiac trunk ostium with a bare metal stent and relining with a standard TEVAR endograft.

One patient died within 30 days; the autopsy revealed a retrograde type A dissection with cardiac tamponade. Early outcome is further presented in Table 3.

**Table 3.** Peri-Operative Outcome (Within 30 Days) After Implantation of a Dissection-Specific Stent-Graft in Patients With Chronic Aortic Dissection (N=30).

Peri-operative 30-day outcome and morbidity	N (%)
Technical success	29 (96.7%)
Mortality	1 (3.3%)
Spinal cord ischemia	2 (6.6%)
Temporary	1
Permanent	1
Cerebrovascular events	1 (3.3%)
CVA	1
TIA	0
Unplanned reinterventions	0
Early dSINE	0
Dissection Propagation (antegrade or retrograde) / rupture <30 days	1 (3.3%)

CVA, cerebrovascular event; TIA, transient vascular accident; dSINE, distal stent-graft induced new entry.

**Figure 3.**
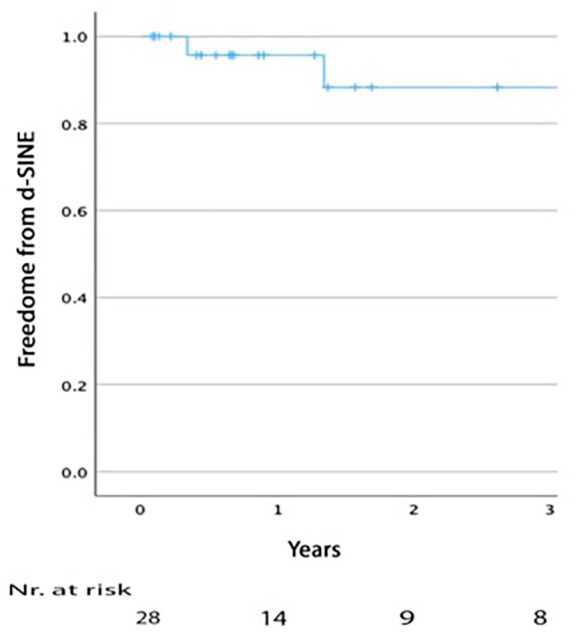
Kaplan–Meier analysis of distal stent-graft induced new entry rate after endovascular treatment of chronic type B dissection with a dissection-specific stent-graft.

**Figure 4.**
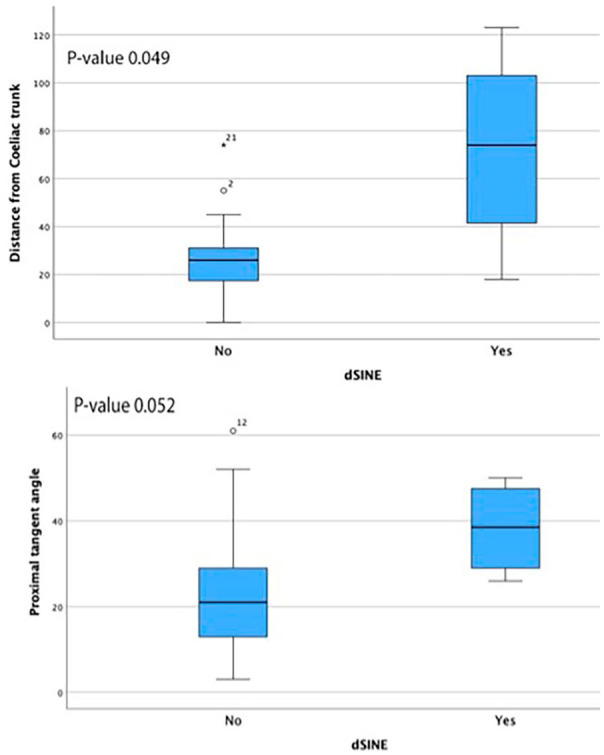
A boxplot displaying the distribution of (a) distance from distal stent graft to coeliac trunk and (b) proximal tangent angle in cases where distal stent-graft induced new entry (dSINE) developed versus non-dSINE patients.

Four patients developed dSINE during follow-up, at intervals of 0.3, 1.3, 4.4, and 5.3 years. Three were asymptomatic, while one presented after 0.3 years with true lumen collapse distal to the dSINE. Of these, 3 were treated with stent-graft extension, 2 with Cook Zenith TX2, and the other with DSSG. Kaplan-Meier estimated freedom from dSINE at 1 and 3 years was 95.6% (SE 0.043) and 89% (SE 0.081), respectively (Figure 3). When assessing anatomical variables associated with dSINE development, the median distance between CT and distal edge of the stent was 74mm (range 18-123) in the dSINE group versus 26mm (range=0-74) in the non-dSINE group (p=0.049). The median aortic angulation at the distal landing zone was 38.5° (range 26-50) in the dSINE group compared with 21° (range 3-61) in the non-dSINE group (p=0.052), Figure 4. Other variables, including connective tissue disease, oversizing, and false lumen thrombosis, were nonsignificant.

Reinterventions > 30-day were performed in 14 patients (50%) with a median reintervention time of 17.8 months (range 0.54-51.1), n=5(18%) were performed for dilatation of the distal (nontreated) thoracoabdominal dissection with fenestrated/branched F-B/EVAR extension of the DSSG. Twelve re-interventions were performed in relation to the previously treated aortic dissection, with false lumen plug n=5 (18%), TEVAR extension n=5 (18%), relining of type 3 endoleak (EL) was performed in n=1 (3.6%) and coiling of type II EL in n=1 (3.6%). In total n=9 (32%) patients had distal reintervention (5 F-B/EVAR and 4 TEVAR). There were two late deaths that were not aortic-related, Table 4. Aortic remodeling and false lumen thrombosis rate are presented in Table 5.

**Table 4.** Long-Term (>30 Days) Clinical Outcomes After Implantation of a Dissection-Specific Stent-Graft in Patients With Chronic Aortic Dissection (N=28).

Long-term (>30 days) clinical outcomes	N (%) or median (range)^a^
Follow-up (months, range)	38.5 (4.3-76.4)
Aortic-related mortality	0
All-cause mortality	2 (7.1%)
Reinterventions ^b^	14 (50%)
FEVAR	5 (18%)
Candy-Plug	5 (18%)
TEVAR	5 (18%)
Distal	4 ^c^
Proximal	1 ^d^
Relining of type III EL	1 (3.6%)
Coil/Plug of type II EL	1 (3.6%)

Values are n (%) or median (range).

a Two patients were excluded from this analysis (1 case of 30-day death, 1 case of technical failure). ^b^ Some had more than one intervention in the same or subsequent procedure. ^c^ Three due to dSINE and one to support Candy-plug at the level of the unsupported fabric ^d^ due to type IA EL. EL, endoleak.

**Table 5.** Long-Term (>30-Day) Radiological Outcome After Implantation of a Dissection-Specific Stent-Graft in Patients With Chronic Aortic Dissection (N=28).

Long-term (>30-day) radiological outcome	N^a^(%) or median (range)
Imaging Follow-up time (months)	13 (1-65.8)
New dSINE≥30 days	4
Median time to new dSINE (months)	35.3 (4.1-65.8)
FL at level of covered descending aorta	
-Not thrombosed	2 (7.1%)
-Partially thrombosed	7 (25%)
-Completely thrombosed	19 (67.9%)
Aneurysm sac behaviour^b^	
Regression>5mm	13 (48%)
Stable	7 (26%)
Expansion>5mm	7 (26%)

Values are n (%) or median (range).

a Two patients were excluded from this analysis (1 case of 30-day death, 1 case of technical failure).

dSINE, distal stent-graft-induced new entry tear; FL, false lumen; ^b^ one missing data, CT scan did not include upper chest.

## Discussion

Endovascular repair of chronic aortic dissection offers a minimally invasive option for a challenging pathology. The development of a dSINE in a fragile dissection membrane however remains a concern. This study shows that implantation of a DSSG with removed distal stents of the TEVAR device, to reduce the radial force exerted on the dissection membrane at the distal landing zone, is feasible and safe in mid-term, and does not preclude future interventions (eg, distal FEVAR) if required during follow-up. A previous computation fluid dynamics (CFD) analysis of the DSSG suggests that the device modifications result in a proximalization of the area where the dissection membrane is exposed to peak shear stress after TEVAR, from the dissection membrane distal to the stent-graft, to the graft-covered distal segment of the TEVAR device, hence potentially reducing the risk for dSINE development.^10^

**Figure 5.**
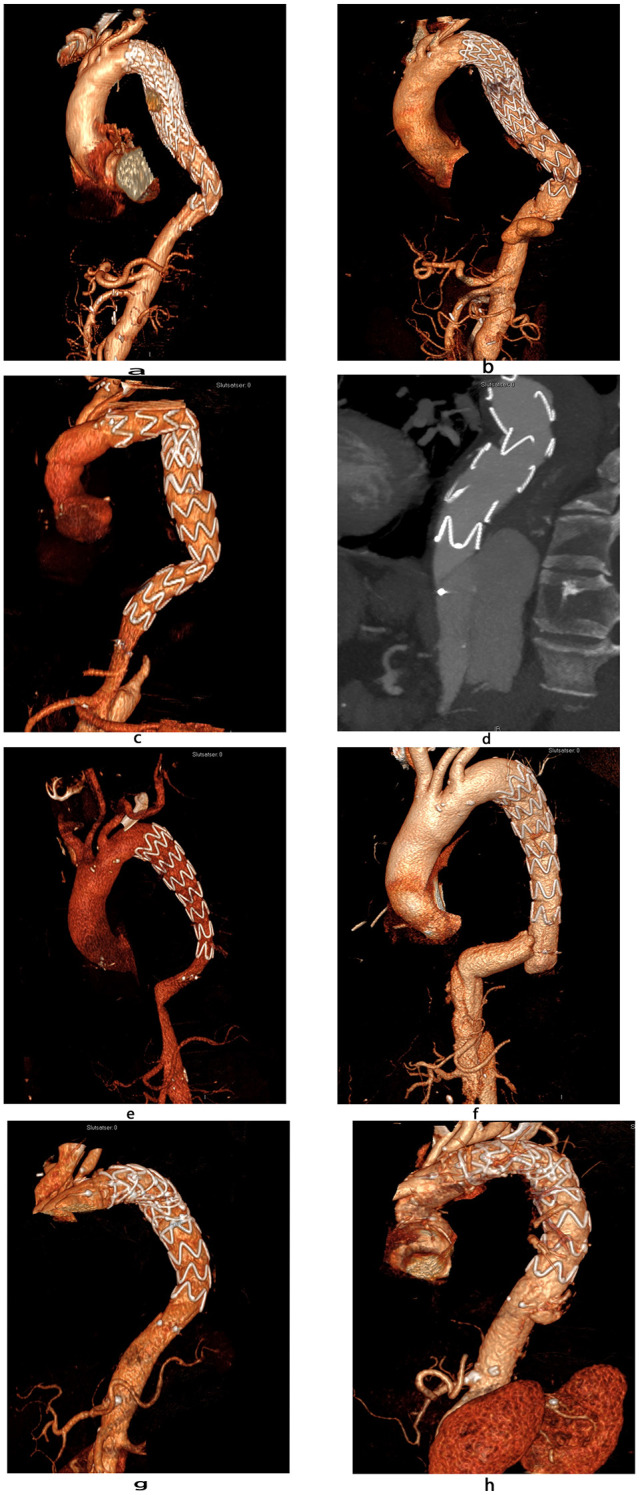
Three-dimensional multi-planar reconstruction of postoperative computed tomography scans of all cases with the development of a distal stent-graft induced new entry after treatment with a dissection-specific stent-graft.

However, the current evaluation of 28 cases treated with DSSG shows that the modified stent-graft does not abolish the risk for dSINE development over time in high-risk patients, with 2 out of 4 patients developing dSINE beyond the 4-year mark following the initial TEVAR procedure. Anatomical evaluation of this cohort suggests that dSINE may be more common when the distal landing zone is in a tortuous aortic segment. Both the “spring back force” (the mechanical tendency of the stent to retrieve its initial straight shape) and the twisting or curving of the aorta might potentially contribute to the development of dSINE due to the increased stresses on the fragile dissection membrane. This aligns with findings from CFD analysis of cases with dSINE post DSSG implantation, where 4 DSSG patients had high wall shear stress (WSS) in the native aorta; however, only 1 patient developed dSINE. In this case, the stent-graft landed in a markedly angulated aortic segment, while the other 3 cases landed in a relatively straight segment. This finding might have implications for DSSG placement and the resultant dynamics in the aortic flow, which could possibly influence the onset of dSINE. The ideal extent of aortic coverage during TEVAR is still debatable, particularly in light of the increasing concern regarding the potential for spinal ischemia with more extensive coverage.^11^ However, the current analysis showed an association between dSINE formation and landing with the distal end of the graft high above the CT (Figure 5). This suggests that it may be beneficial to extend the TEVAR in chronic dissection setting to a straight aortic segment close to the coeliac trunk, aiming to reduce the risk for dSINE.

The commercially available thoracic stent-grafts often taper by 4mm. Endografts tapered >4mm and custom-made devices would be beneficial when standard devices still result in significant distal oversizing. The impact of distal oversizing (dOS) on the dissection membrane in the DSSG design should intuitively be small, considering that a nonsupported polyester graft is inserted in the true lumen without the radial force of the stent against the dissection membrane. Generally, measurement of the distal landing zone in chronic dissection and sizing of the graft in this section is challenging considering the often elliptic shape of the true lumen. This study revealed an unexpectedly high level of oversizing when comparing the graft size to the mean size of the true lumen. Although we did not identify any substantial correlation between dOS and the occurrence of dSINE, 2 out of 4 dSINE cases had a dOS over 30%. Among various considerations, optimizing distal graft diameter with less oversizing (to <10%) may be an important modification to reduce the risk for dSINE also in the DSSG setting.

In the current cohort, high technical success was achieved with the DSSG, and a significant proportion of patients exhibited a favorable aortic diameter change, with complete or partial false lumen thrombosis in most cases. Re-interventions were required over time in n=14 (50%) patients and underline the need for continuous follow-up after TEVAR in patients with chronic aortic dissection. While TEVAR often results in positive aortic remodeling in the thoracic aorta, continued expansion may occur in the dissected para-visceral and abdominal aortic segment, requiring distal extension with fenestrated or branched EVAR.^2^

The high percentage of reintervention within a median of 38 months could be affected by the patient selection for DSSG. Some patients had connective tissue disorders, and others had established dSINE after previous aortic procedures. Both cohorts could potentially have a more fragile septum that is more prone to dSINE formation after TEVAR.

Extensive coverage until the proximity to the CT demonstrated favorable outcomes in relation to dSINE occurrence and has the potential as well to reduce the necessity for future reintervention in this high-risk patient subset. However, when treating patients with chronic dissection, staging surgery with repeat interventions when necessary is part of the treatment strategy. Thus, reintervention for progressive aortic dilatation distal to the previously treated segment should not be regarded as treatment failure. In the current experience, the presence of a DSSG with an unsupported distal graft segment in the TEVAR device did not present any issues during a fenestrated or branched extension of the TEVAR. The use of a candy plug at the level of the unsupported fabric may, however, be more controversial, as the radial force of the false lumen occluder may compromise true lumen integrity. To address this issue, we extended the DSSG with another stent graft to ensure that the radial force of the stent-graft in the true lumen was equal or superior to that of the candy plug.

The limitations of this study include the relatively small sample size, the single-center, nonrandomized design, and the lack of a control group. In addition, the study has a substantial loss of follow-up, mainly due to distal reinterventions. Therefore, future research involving larger and more diverse patient cohorts will be beneficial in substantiating these findings and further understanding the potential of DSSG for TEVAR treatment of chronic aortic dissection. Longer-term follow-up is required to provide more information about the durability of the DSSG and late dSINE occurrence.

## Conclusion

The use of a novel DSSG with low radial force for TEVAR in the setting of chronic aortic dissection is safe and feasible, with 30-day and mid-term remodeling outcome comparable with standard TEVAR. While the reduced radial force of the DSSG has been previously associated with a lower short-term risk of dSINE, it does not eliminate the long-term risk for dSINE development. Notably, new entries tend to occur, particularly in cases where the distal landing zone is in a tortuous aortic segment and is not close to the coeliac artery.
